# SET oncoprotein accumulation regulates transcription through DNA demethylation and histone hypoacetylation

**DOI:** 10.18632/oncotarget.15818

**Published:** 2017-03-01

**Authors:** Luciana O. Almeida, Marinaldo P.C. Neto, Lucas O. Sousa, Maryna A. Tannous, Carlos Curti, Andreia M. Leopoldino

**Affiliations:** ^1^ Department of Clinical Analyses, Toxicology and Food Sciences, School of Pharmaceutical Sciences of Ribeirão Preto, University of São Paulo, Ribeirão Preto, SP, Brazil; ^2^ Department of Physics and Chemistry, School of Pharmaceutical Sciences of Ribeirão Preto, University of São Paulo, Ribeirão Preto, SP, Brazil; ^3^ Department of Genetics and Evolution, Federal University of São Carlos, São Carlos, SP, Brazil; ^4^ CEPID-FAPESP, Center for Cell Based Therapy, Hemotherapy Center of Ribeirão Preto, Ribeirão Preto, SP, Brazil

**Keywords:** chromatin, HNSCC, SET protein, histone acetylation, active DNA demethylation

## Abstract

Epigenetic modifications are essential in the control of normal cellular processes and cancer development. DNA methylation and histone acetylation are major epigenetic modifications involved in gene transcription and abnormal events driving the oncogenic process. SET protein accumulates in many cancer types, including head and neck squamous cell carcinoma (HNSCC); SET is a member of the INHAT complex that inhibits gene transcription associating with histones and preventing their acetylation. We explored how SET protein accumulation impacts on the regulation of gene expression, focusing on DNA methylation and histone acetylation. DNA methylation profile of 24 tumour suppressors evidenced that SET accumulation decreased DNA methylation in association with loss of 5-methylcytidine, formation of 5-hydroxymethylcytosine and increased TET1 levels, indicating an active DNA demethylation mechanism. However, the expression of some suppressor genes was lowered in cells with high SET levels, suggesting that loss of methylation is not the main mechanism modulating gene expression. SET accumulation also downregulated the expression of 32 genes of a panel of 84 transcription factors, and SET directly interacted with chromatin at the promoter of the downregulated genes, decreasing histone acetylation. Gene expression analysis after cell treatment with 5-aza-2′-deoxycytidine (5-AZA) and Trichostatin A (TSA) revealed that histone acetylation reversed transcription repression promoted by SET. These results suggest a new function for SET in the regulation of chromatin dynamics. In addition, TSA diminished both SET protein levels and SET capability to bind to gene promoter, suggesting that administration of epigenetic modifier agents could be efficient to reverse SET phenotype in cancer.

## INTRODUCTION

Epigenetics refers to heritable changes in gene expression not related to changes in the DNA sequence [1]; they occur primarily through DNA methylation and histone post-translational modifications. Epigenetic changes have been associated with specific patterns of gene expression and are early events in different cancer types, including head and neck squamous cell carcinoma (HNSCC) [2]. In mammals, DNA methylation occurs predominantly at CpG dinucleotides. DNA methyl transferase (DNMT) transfers a methyl group to cytosine, generating 5-methylcytosine [3]. Low methylation levels within the promoter region have been associated with either gene activation or chromosome instability [4].

Lack or reduction of DNMT activity implies gradual and passive loss of DNA methylation, so that DNMTs inhibition is an important mechanism of the passive DNA demethylation process. DNA demethylation also occurs through an active mechanism, independent of cell division, which is mediated by enzymes of the TET family. These enzymes firstly modify methylated cytosine, which is subsequently replaced by the DNA repair machinery [5, 6].

Changes in chromatin dynamics is an early abnormality described in cancer [7]. The basic unit of chromatin is the nucleosome, which is formed by DNA and histone proteins [8]. Histones undergo many modifications in their N-terminal tails, among which lysine acetylation is the most studied one. Histone deacetylases (HDAC) remove the acetyl radical present in the lysine residues of histones, whereas histone acetyltransferases (HAT) add acetyl radicals to those positions [1]. The chromatin organization results from post-translational modifications of histone tails, DNA methylation patterns, and nucleosome positioning. In cancer cells, chromatin remodeling, methylation signatures, and consequently, gene expression profiles, become disturbed [7].

SET oncoprotein (I2PP2A or TAF-1β), which is accumulated in HNSCC [9], is involved in many aspects related to chromatin structure. SET protein is characterized as (*i*) an histone chaperone required for chromatin transcription [10, 11], (*ii*) an important regulator of chromosome condensation [12], (*iii*) a member of the Inhibitor of Histone Acetyltransferase (INHAT) complex that inhibits histone acetylation by binding to and masking histone acetyltransferases targets [13], and (*iv*) an inhibitor of active DNA demethylation [14]. In this context, it is unknown whether SET accumulation in cells either modifies specific targets or is associated with transcriptional repression or activation. Here, we addressed this issue. Overall, we demonstrated that SET promotes a global DNA demethylation in HNSCC cells, through active signaling, increasing TET1 and 5-hydroxymethylcytosine levels. Demethylation is not always followed by reactivation of gene expression.

## RESULTS

### SET accumulation is associated with global loss of DNA methylation in HNSCC

**Figure 1:**
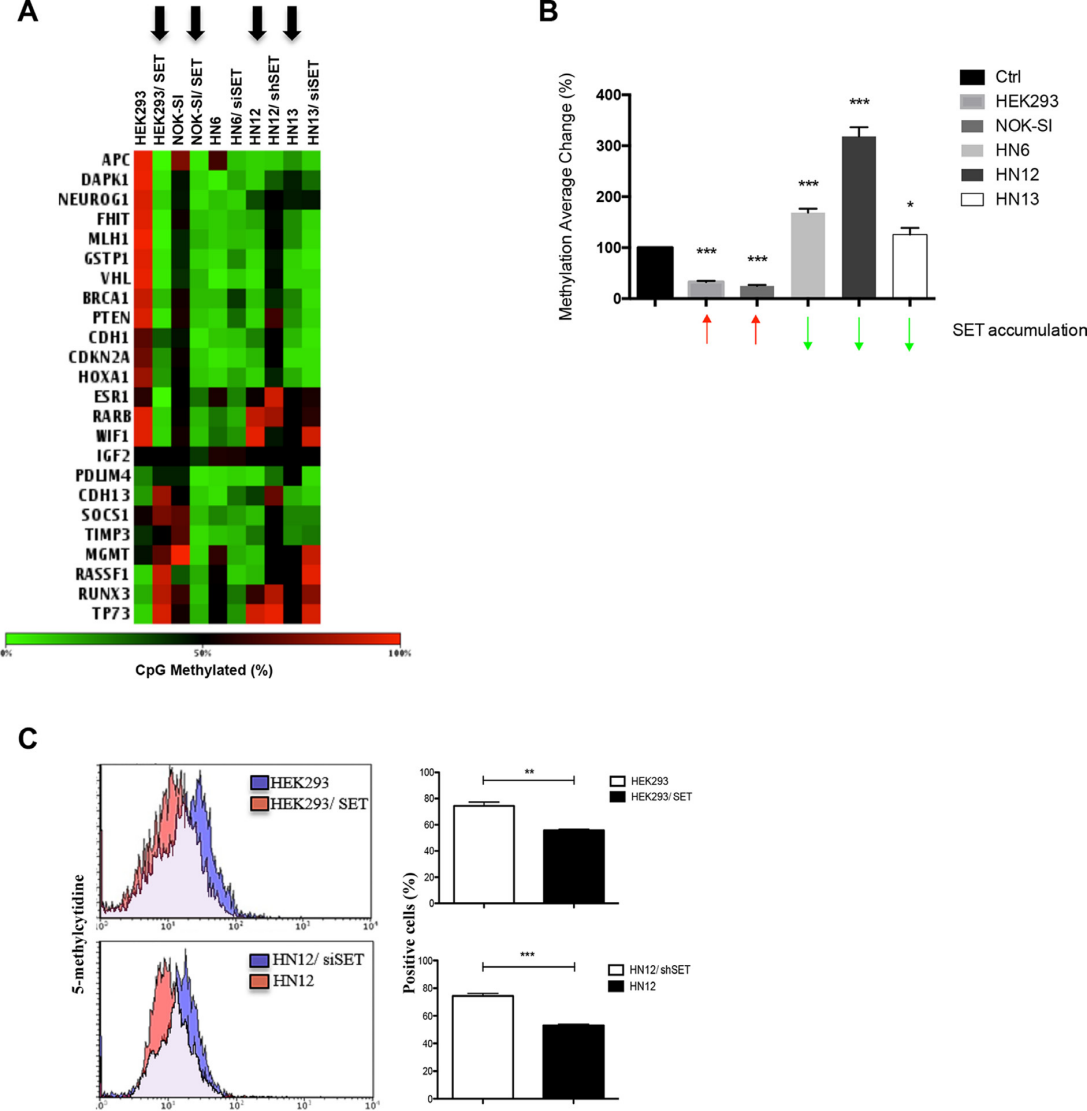
SET protein promotes loss of DNA methylation (**A**) Normal cell lines HEK293 and NOK-SI – control and overexpressing SET protein (HEK293/SET and NOK-SI/SET) –, and HNSCC cell lines HN6, HN12, and HN13 – control and with SET knockdown (HN6siSET, HN12siSET, and HN13siSET, respectively) – were used to assess the methylation profile of tumor suppressor genes. An empty vector and the siControl AllStar siRNA negative control oligonucleotide were used as controls for SET overexpression and SET knockdown, respectively. DNA was extracted with phenol:chloroform and the DNA methylation profile was assessed using EpiTect Methyl PCR array (Qiagen), according to the manufacturer's instructions. After qPCR assay, data were analyzed using PCR array Data Analysis Software (Qiagen). The heat map indicates hypomethylated genes in green and hypermethylated genes in red. (**B**) Graphic representation of the methylation average changes in cells after SET knockdown or overexpression. We calculated the ratio of changes in CpG methylation levels between control and SET up- or downregulated samples. (**C**) Flow cytometry assay was used to quantify global methylation levels though 5-methylcytidine accumulation in HEK293, HEK293/SET, HN12, and HN12siSET cells. Cells without antibody staining were used as negative control (no staining). Assays were performed in triplicate. *(*p* < 0.05), **(*p* < 0.01), and ***(*p* < 0.001).

To investigate how SET protein accumulation influences the epigenetic control of gene expression, we analysed the DNA methylation profile in two non-tumour cell lines, HEK293 and NOK-SI, with and without SET overexpression (HEK293/SET and NOK-SI/SET, respectively), and three HNSCC cell lines, HN6, HN12, and HN13, which present SET constitutively accumulated [15], with and without SET knockdown (HN6siSET, HN12siSET, and HN13siSET, respectively). As the oncogene SET is a potent inhibitor of the tumour suppressor PP2A [16, 17], and inhibits other tumour suppressors such as PTEN [18] and TP53 [19], we examined how SET accumulation affects DNA methylation using a panel of known tumour suppressor genes by means of EpiTect Methyl qPCR arrays (Figure 1A). Notably, SET protein accumulation, a common event in HNSCC [15], promoted loss of DNA methylation (Figure 1A - black arrows). Next, we analysed the average methylation change to know how much the changes in SET levels would influence the methylation profile. We calculated the ratio of CpGs methylated between control and up- or downregulated SET samples (Figure 1B). SET-accumulating cells displayed decreased average DNA methylation (Figure 1B - red arrows), while the HNSCC cell lines with knocked down SET displayed increased average DNA methylation (Figure 1B – green arrows); these findings suggest that SET accumulation influences DNA methylation.

To confirm these results, we analysed the cellular levels of 5-methylcytidine, which is commonly used to assess the full genome methylation profile [20]. We selected the HN12 cell line – which exhibited the most marked change in average DNA methylation after SET knockdown (Figure 1B) – and the non-tumour cell line HEK293 (Figure 1C) for the assay. Compared with HEK293 cells, HEK293/SET cells significantly lost 5-methylcytidine, while HN12 cells exhibited low 5-methylcytidine levels. As expected, HN12siSET cells had increased 5-methylcytidine levels. These results indicate that SET modulates DNA methylation levels in HNSCC.

**Figure 2:**
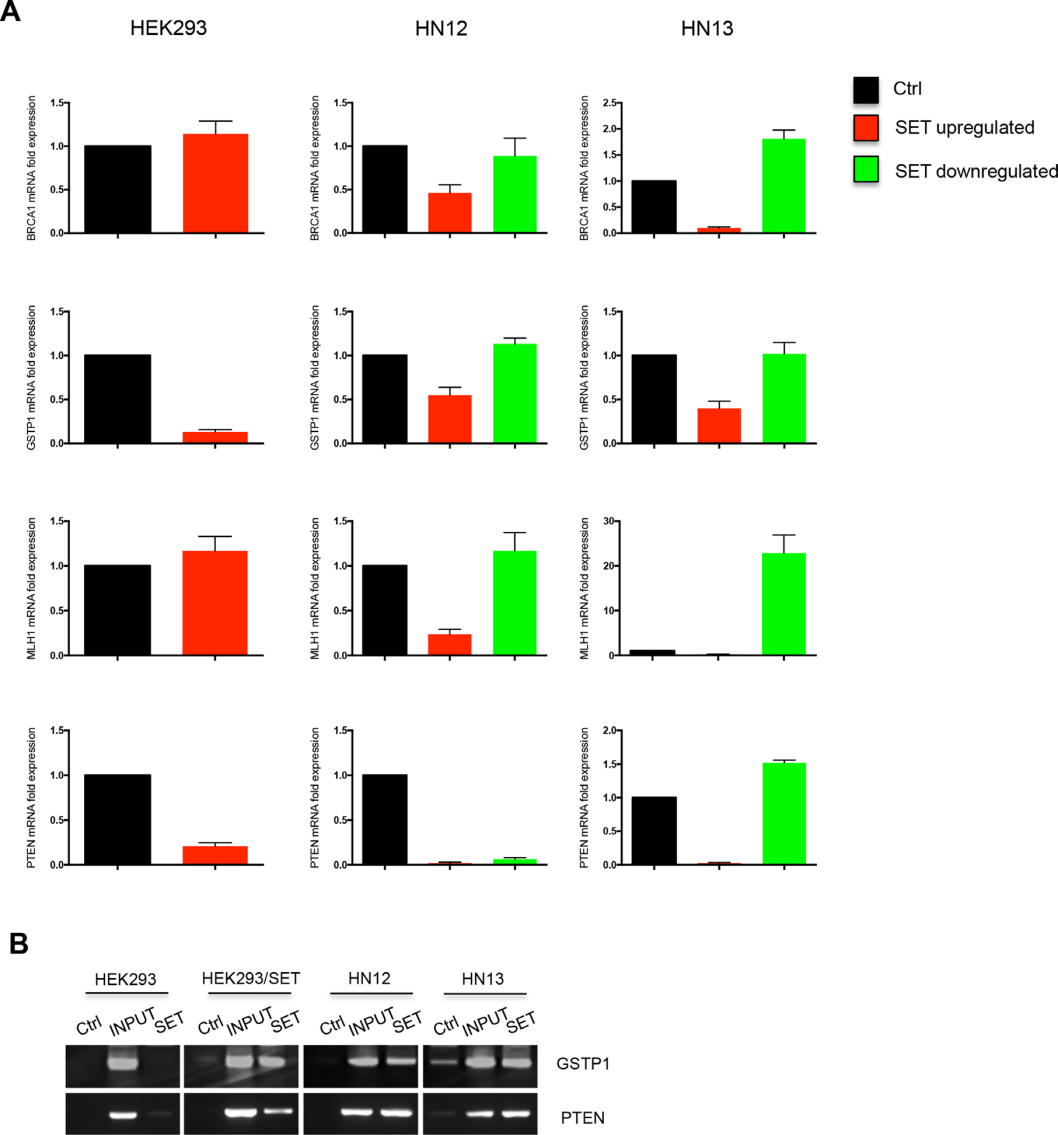
Loss of DNA methylation driven by SET protein does not necessarily activate gene expression (**A**) Quantitative PCR was performed in HEK293, HEK293/SET, HN12, HN12siSET, HN13, and HN13siSET cells to evaluate whether tumor suppressor genes expression was affected by DNA methylation. *BRCA1*, *MLH1*, and *PTEN* were assessed through TaqMan probes and *GSTP1* through SybrGreen primers. Graphics represent relative quantification of experiments performed in triplicate through 2^−ΔΔCt^ method. CDNA from HEK293 was used as calibrator and GAPDH and β-globin primers were used as housekeeping. (**B**) Chromatin immunoprecipitation assay was performed to identify whether SET interacts with *GSTP1* and *PTEN* promoters. Conventional PCR using DNA immunoprecipitated with antibody against SET was performed in triplicate. Ctrl lanes represent samples immunoprecipitated with anti-IgG antibody; INPUT samples consist of total DNA, and SET lanes refer to DNA immunoprecipitated with anti-SET antibody.

Given that DNA methylation is an epigenetic mechanism for silencing gene expression, we performed qPCR to assess the expression levels of four genes that were hypomethylated by SET accumulation: *BRCA1*, *GSTP1*, *MLH1*, and *PTEN* (Figure 2A). They were analysed in HEK293, HN12, and HN13 cells. SET overexpression in HEK293 cells upregulated *BRCA1* and *MLH1* expression, but downregulated *GSTP1* and *PTEN* expression (Figure 2A – HEK293 column). Compared with the basal expression levels in the non-tumour cell line HEK293, used as calibrator, the expression levels of all four genes were lowered in HN12 and HN13 tumour cells (Figure 2A – red bars) but increased after SET knockdown (Figure 2A – green bars). Thus, high SET levels in tumour cells directly correlated with diminished expression of these genes. This finding was unexpected because SET accumulation is associated with loss of DNA methylation, which activates the expression of genes that are typically silenced by methylation [21]. A plausible explanation is that DNA methylation responds differently to the gene expression control machinery due to the interaction with other factors, including histone modifiers [22]. In addition, SET negatively regulates the expression of genes involved in cellular detoxification [23] and also participates as a subunit of the inhibitor of histone acetyltransferases complex that represses transcription [24], suggesting that the SET controls gene expression through a DNA methylation-independent mechanism. Considering that SET, as a member of the INHAT complex, can directly bind to histones at promoters, to prevent acetylation [13, 24], we assessed the SET-promoter interaction using the chromatin immunoprecipitation assay (ChIP) for two genes whose expressions were decreased by SET accumulation: *GSTP1* and *PTEN* (Figure 2A). Both genes demonstrated association with SET at the promoter in all SET-accumulating cells, but not in HEK293 cells, which do not accumulate SET (Figure 2B).

### Active DNA demethylation is the mechanism activated by SET for the loss of methylation

**Figure 3:**
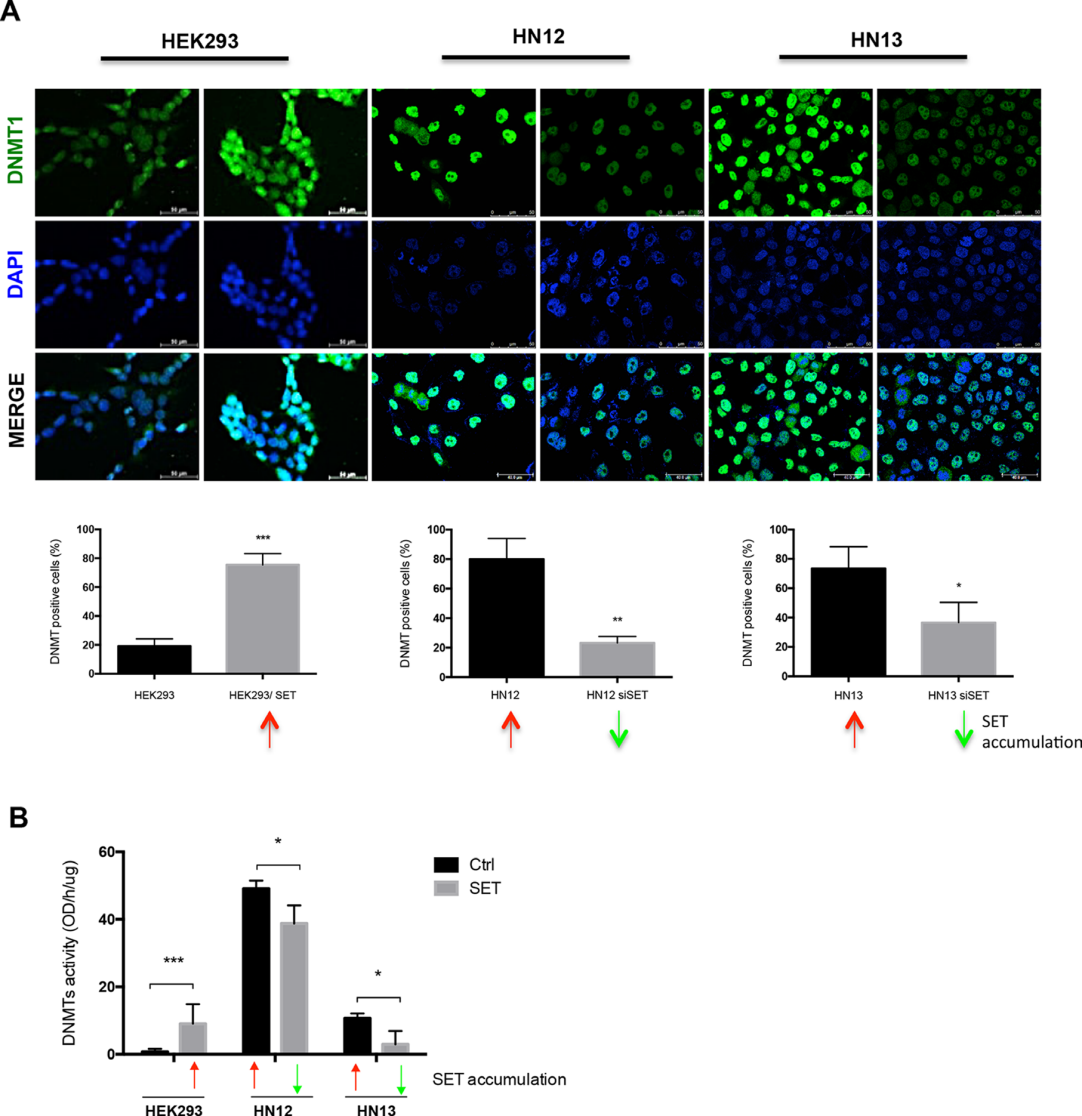
DNA methyltransferases are not involved in DNA demethylation process mediated by SET accumulation . (**A**) Immunofluorescence assay was performed in HEK293, HEK293/SET, HN12, HN12siSET, HN13, and HN13siSET cells to determine the levels of DNMT1, the enzyme responsible for DNA methylation maintenance. Cells from five fields were measured using ImageJ software. Graphics represent the percentage of DNMT1-positive cells. Red arrows indicate SET accumulation; green arrows indicate SET downregulation. (**B**) DNA methyltransferases enzymatic activity assay using nuclear protein extract from cells; this assay was perfomed using EpiSeeker DNMT Activity Quantification Kit (Abcam), according to the manufacturer's protocol. Graphic represents experiments performed in triplicate. *(*p* < 0.05),**(*p* < 0.01) and ***(*p* < 0.001).

DNMTs are responsible for the establishment and maintenance of DNA methylation. In this regard, DNMT inhibition is the main mechanism of passive DNA demethylation [5]. Given that DNA methylation pattern is maintained through cell division by DNMT1 activation [25], we assessed whether SET overexpression reduces DNMT1 levels in HEK293, HN12, and HN13 cells, using an immunofluorescence assay. Remarkably, all SET-accumulating cells exhibited increased DNMT1 levels, and SET knockdown decreased DNMT1 levels (Figure 3A). As SET accumulation is associated with reduced DNA methylation, we would expect that SET overexpression downregulated DNMT1; then, we also assessed DNMT activity using the same cell lines. Accordingly, SET accumulation and downregulation were associated with augmented and diminished DNMT activity, respectively (Figure 3B).

**Figure 4:**
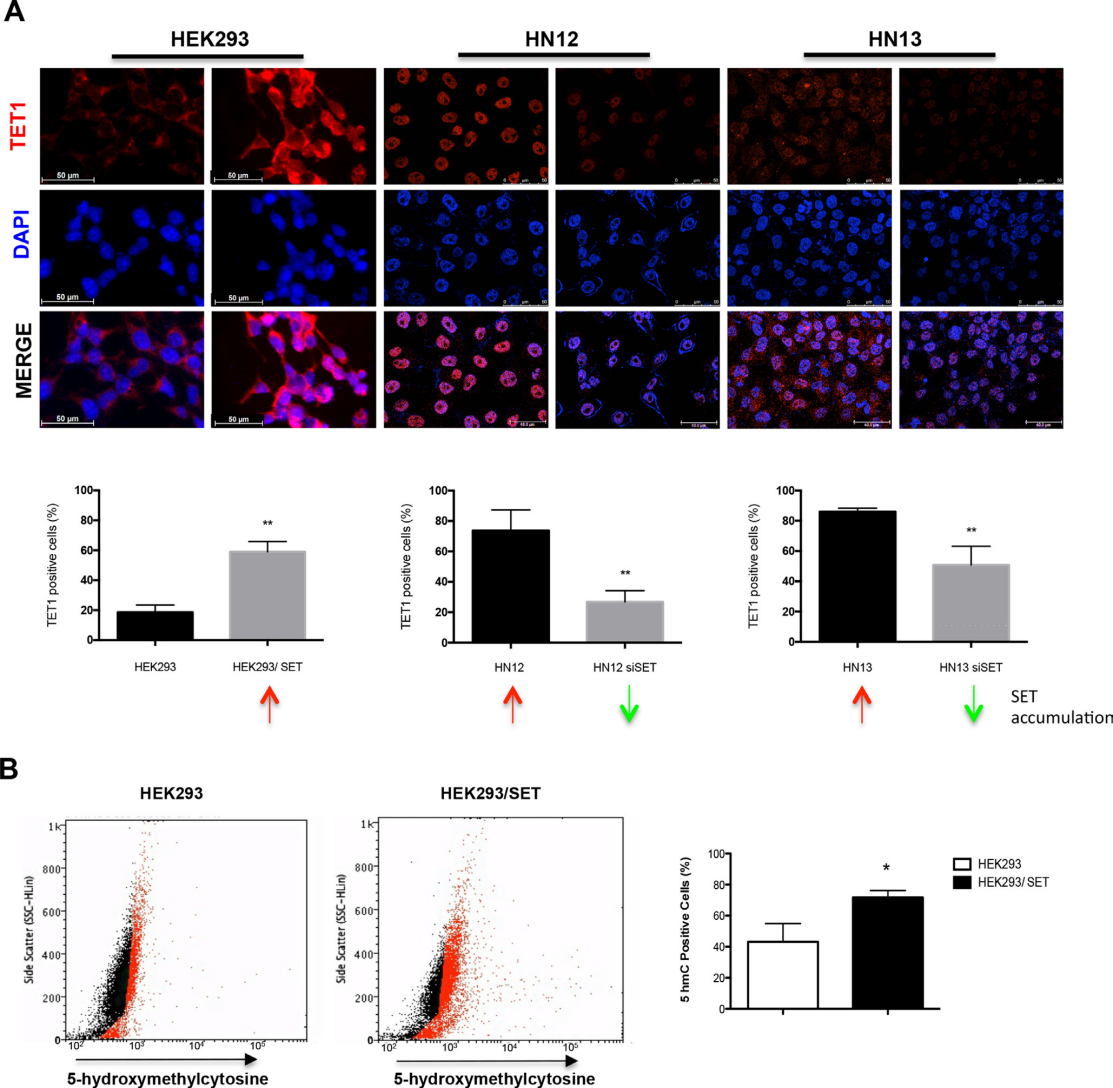
Loss of DNA methylation is associated with increased levels of TET1 and 5-hydroxymethylcytosine (**A**) Immunofluorescence assay using anti-TET1 antibody was performed in HEK293, HEK293/SET, HN12, HN12siSET, HN13, and HN13siSET cells to assess active DNA demethylation process. Cells from five fields were measured using ImageJ software. Graphics represent the percentage of TET1-positive cells. Red arrows indicate SET accumulation; green arrows indicate SET downregulation. (**B**) Flow cytometry assay was performed to measure the levels of 5-hydroxymethylcytosine in HEK293 and HEK293/SET cells. The respective unstained cells were used as negative controls; dead cells were excluded from the plot. The assay was performed in triplicate. *(*p* < 0.05) and **(*p* < 0.01).

DNA demethylation can also occur through an active mechanism. Active DNA demethylation is an enzymatic process that removes the methyl group from 5-methylcytosine by breaking carbon-carbon bonds [26]. One of the active DNA demethylation mechanisms involves the oxidation of 5-methylcytosine, which is converted into 5-hydroxymethylcytosine by TET enzymes [6]. Here, we used an immunofluorescence assay to determine the TET1 protein levels in HEK293, HN12, and HN13 cells. We detected high TET1 levels in SET-accumulating cells and decreased TET1 levels after SET knockdown (Figure 4A).

Since active demethylation converts 5-methylcytosine into 5-hydroxymethylcytosine [5], we also explored this product by flow cytometry. To assess exclusively the effect of the SET protein in the process, we selected the non-tumour cell line HEK293 overexpressing it (Figure 4B). Consistent with the TET1 levels, 5-hydroxymethylcytosine levels increased in HEK293/SET cells, suggesting that SET accumulation promotes active DNA demethylation by elevating the levels of TET1, which subsequently controls the oxidation of 5-methylcytosine and its conversion into 5-hydroxymethylcytosine.

### SET accumulation downregulates gene expression through a histone deacetylation-dependent mechanism

**Figure 5:**
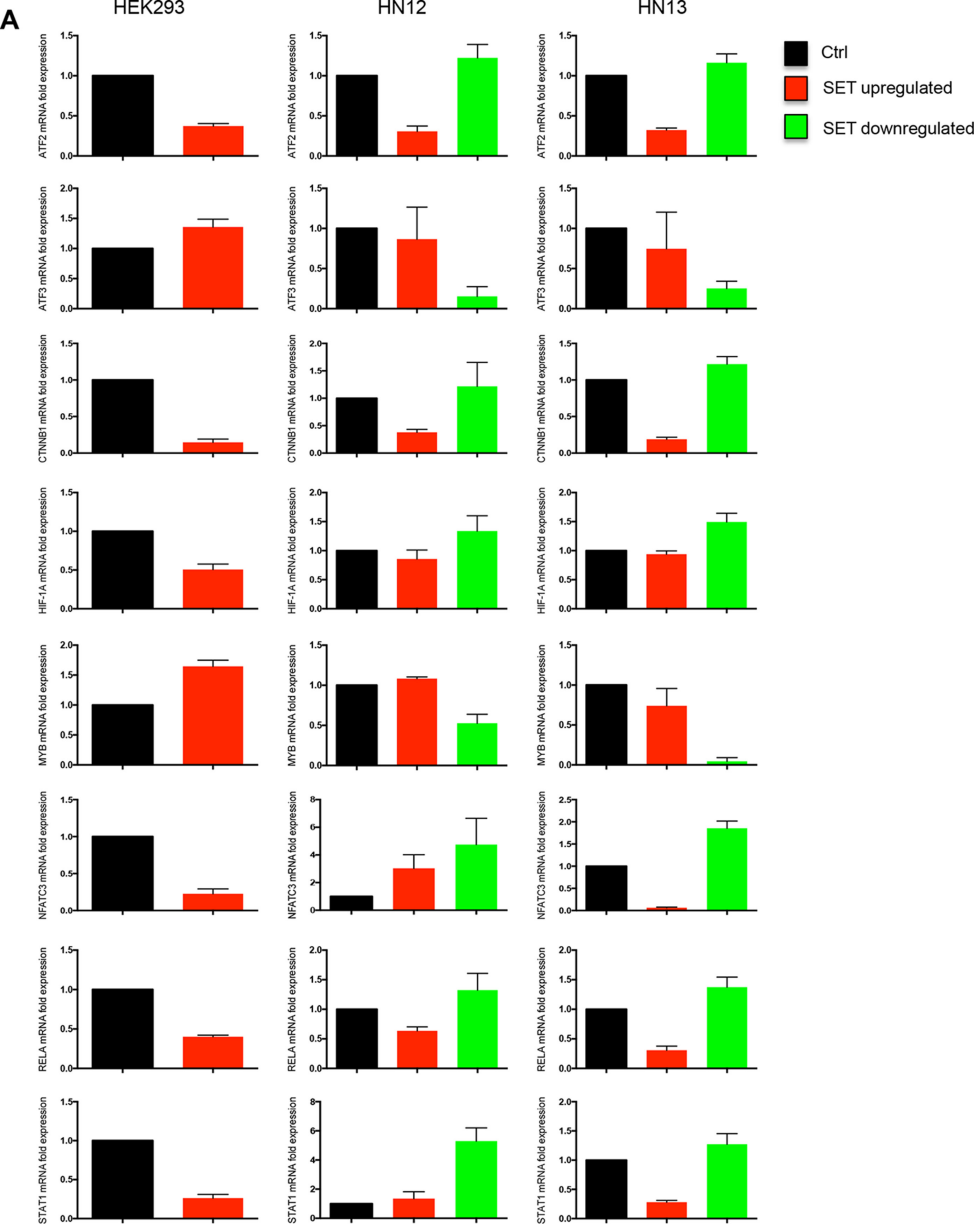
Gene expression of human transcription factors is downregulated through SET protein Quantitative real time PCR was performed to validate the gene expression of six transcription factors downregulated by SET (*ATF2*, *CTNNB1*, *HIF-1A*, *NFATC3*, *RELA*, and *STAT1*) and two transcription factors upregulated by SET (*ATF3* and *MYB*), using SybrGreen primers in HEK293, HEK293/SET, HN12, HN12siSET, HN13, and HN13siSET cells. Graphics represent relative quantification of experiments performed in triplicate through the 2^−ΔΔCt^ method. CDNA from HEK293 cells was used as calibrator; GAPDH and β-globin primers were used as housekeeping.

**Figure 6:**
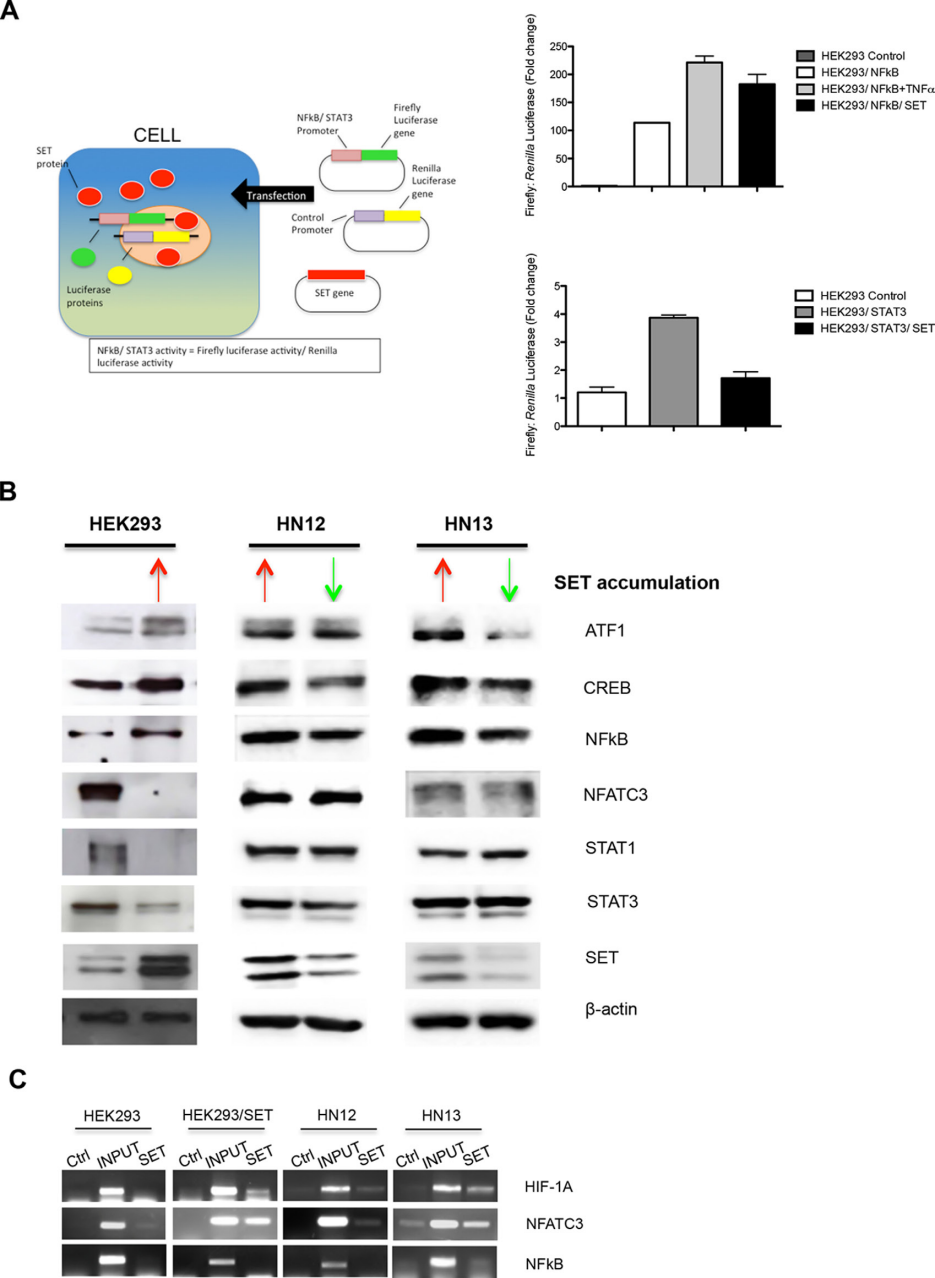
SET contributes to low gene expression levels through chromatin association (**A**) Luciferase reporter assay was performed to quantify NFkB and STAT3 expression induced by SET accumulation. HEK293 cells were transfected with plasmids containing cDNA to overexpress SET, and plasmids with gene promoter region for *NFkB* (PGL3-NFkB-Luc) and *STAT3* (PGL3-STAT3-Luc). (**B**) Western blot assay was used to assess whether downregulation of gene expression could be controlling protein levels. Total proteins were obtained from HEK293, HEK293/SET, HN12, HN12siSET, HN13, and HN13siSET cells. ATF1, CREB, NFkB, NFATC3, STAT1, STAT3, SET, and β-actin proteins were analyzed. Red arrows indicate SET accumulation; green arrows indicate SET downregulation. (**C**) Chromatin immunoprecipitation assay was performed to assess whether SET is able to downregulate the expression of transcription factor genes through binding to the promoter. DNA of the respective cells was immunoprecipitated with anti-SET antibody, and PCR was performed using primers for HIF-1A, NFATC3, and NFkB promoters. Ctrl lanes represent samples immunoprecipitated using anti-IgG antibody; INPUT samples consist of total DNA; SET lanes refer to DNA immunoprecipitated with anti-SET antibody.

Although our finding have evidenced that SET promotes DNA demethylation through a well-characterized active mechanism, involving TET1 (Figure 4A) and 5-hydroxymethylcytosine (Figure 4B) [5], the present study also evidenced a downregulated gene expression driven by SET accumulation (Figure 2A). SET is a member of the INHAT complex that inhibits gene transcription through association with histones, thus preventing their acetylation by blocking the association histones-transcriptional coactivators [24]. To confirm the effects of SET accumulation on gene expression regulation, we performed a screening in HEK293 and HEK293/SET cells by employing a qPCR array system designed for a panel of 84 transcription factors (Table 1). We found that 42% of the genes were downregulated and 19% were upregulated. For validation of the results, we performed qPCR for 8 genes, whose expressions were altered, in HEK293, HEK293/SET, HN12, HN12siSET, HN13, and HN13siSET cells (Figure 5). SET accumulation downregulated six genes (*ATF2*, *CTNNB1*, *HIF-1A*, *NFATC3*, *RELA*, and *STAT3*) and upregulated two genes *(ATF3* and *MYB*) (Figure 5 - red bars). Overall, SET knockdown promoted the opposite effect (Figure 5 - green bars). We also assessed the effects of SET accumulation on the regulation of expression via promoters of two transcription factors, *NFkB* and *STAT3*, by using a luciferase reporter assay. *NFkB*, which was upregulated as assessed by qPCR array analysis, presented increased levels in SET-accumulating cells (Figure 6A - above). In agreement with the qPCR array results, *STAT3* presented decreased levels in SET-accumulating cells (Figure 6A, below). These results confirm our previous findings that augmented SET levels control the expression of genes associated with tumorigenesis in HNSCC.

**Table 1:** Human transcription factors qPCR array performed in HEK293 and HEK293/SET cells

*Gene ID*	*mRNA Fold Expression*	*Expression Levels*	*Gene ID*	*mRNA Fold Expression*	*Expression Levels*
AR	0.3528	decreased	MEF2B	0.7254	ns
ARNT	1.0769	ns	MEF2C	0.9375	ns
ATF1	1.523	ns	MYB	5.3034	increased
ATF2	0.4435	decreased	MYC	0.3888	decreased
ATF3	8.6154	increased	MYF5	-	-
ATF4	1.4814	ns	MYOD1	1.4016	ns
CEBPA	1.3537	ns	NFAT5	0.4225	decreased
CEBPB	0.3071	decreased	NFATC1	0.2906	decreased
CEBPG	1.5877	ns	NFATC2	0.4404	decreased
CREB1	2.9627	increased	NFATC3	0.4331	decreased
CREBBP	2.5614	increased	NFATC4	2.095	increased
CTNNB1	0.3136	decreased	NFKB1	2.5437	increased
DR1	1.1072	ns	NFYB	-	-
E2F1	0.3455	decreased	NR3C1	0.6359	ns
E2F6	2.1095	increased	PAX6	0.9506	ns
EGR1	2.1095	increased	POU2AF1	-	-
ELK1	1.0259	ns	PPARA	0.7056	ns
ESR1	1.4015	ns	PPARG	2.2925	increased
ETS1	1.2032	ns	RB1	1.8881	ns
ETS2	1.5125	ns	REL	0.4411	decreased
FOS	1.2457	ns	RELA	0.4196	decreased
FOXA2	0.4375	decreased	RELB	2.4401	decreased
FOXO1	1.4711	ns	SMAD1	0.4483	decreased
GATA1	-	-	SMAD4	1.4112	ns
GATA2	0.4343	decreased	SMAD5	5.1228	increased
GATA3	0.2012	decreased	SMAD9	0.4887	decreased
GTF2B	3.046	increased	SP1	2.6517	increased
GTF2F1	1.4917	ns	SP3	1.9412	ns
HAND1	0.4284	decreased	STAT1	0.4872	decreased
HAND2	0.9246	ns	STAT2	0.2826	decreased
HDAC1	1.6323	ns	STAT3	0.4722	decreased
HIF1A	0.4108	decreased	STAT4	1.7739	ns
HNF4A	1.4015	ns	STAT5A	0.3136	decreased
HOXA5	0.4311	decreased	STAT5B	3.2873	increased
HSF1	2.3245	increased	STAT6	1.3918	ns
ID1	1.22	ns	TBP	0.4508	decreased
IRF1	0.4294	decreased	TCF1	-	-
JUN	0.4053	decreased	TCF7L2	1.4015	ns
JUNB	0.6359	ns	TFAP2A	0.3384	decreased
JUND	0.4165	decreased	TGIF1	2.4913	increased
MAX	0.4313	decreased	TP53	0.4815	decreased
MEF2A	0.6493	ns	YY1	1.3076	ns

mRNA fold change expression was calculated using PCR array Data Analysis Software (Qiagen). Genes with fold expression value below 0.5 were considered downregulated, and genes with fold expression value above 2.0 were considered up-regulated. ns (non-significant).

Next, we assessed whether protein levels of the transcription factors regulated by SET correlate with their respective mRNA levels. Western blotting analysis of six transcription factors (ATF1, CREB, NFkB, NFATC3, STAT1, and STAT3) demonstrated the existence of such correlation. In line with the gene expression data, the ATF1, CREB, and NFkB protein levels were elevated, and the NFATC3, STAT1, and STAT3 protein levels were diminished in SET-accumulating cells (Figure 6B).

Genes in an inactive state exhibit promoters that are hypoacetylated and allow SET binding to chromatin, thereby blocking the access of histone acetyltransferases and preventing gene expression [24]. Given that 42% of the transcription factors were downregulated in HEK293/SET cells, we assessed whether SET protein interacts with chromatin to downregulate their expression. We performed the ChIP assay for the promoter region of *HIF-1A* and *NFATC3*, which were downregulated by SET (Figure 5), and of *NFkB*, which was upregulated by SET (Figure 6A). In SET-accumulating cells (HEK293/SET, HN12, and HN13), SET interacted with *HIF-1A* and *NFATC3* chromatin, but not with *NFkB* chromatin (Figure 6C).

**Figure 7:**
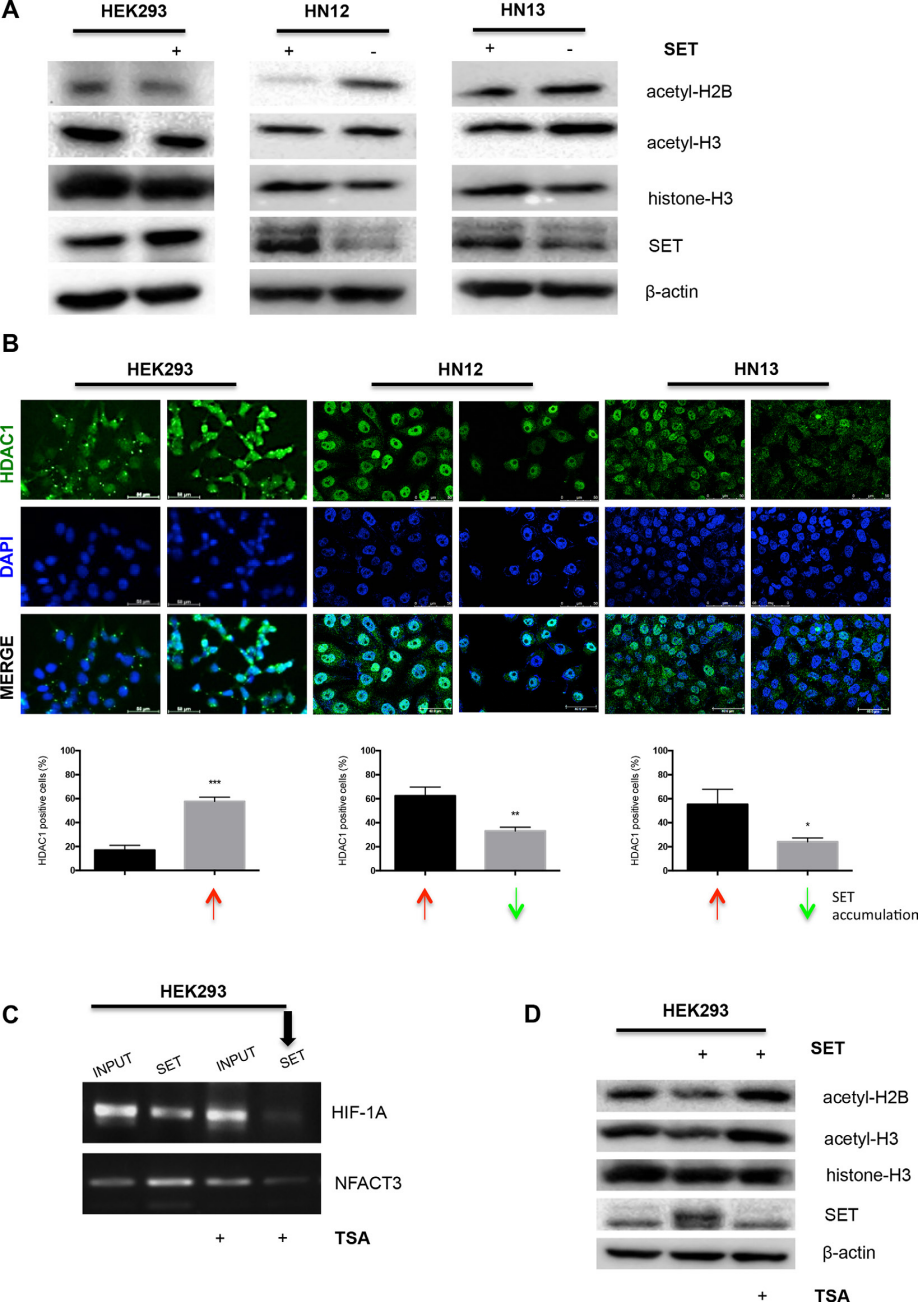
SET may modulate gene expression through hypoacetylation of histones and increase of HDAC1 levels (**A**) Western blot assay was performed by adding antibodies against acetyl-H2B, acetyl-H3, SET, and β-actin to total proteins extracted from HEK293, HEK293/SET, HN12, HN12siSET, HN13, and HN13siSET cells. (**B**) HDAC1 levels were assessed using immunofluorescence assay. Cells from five fields were measured using ImageJ software. Graphics represent the percentage of TET1-positive cells. Red arrows indicate SET accumulation; green arrows indicate SET downregulation. (**C**) To assess whether SET directly associates with sites of hypoacetylated gene promoter, HEK293 and HEK293/SET cells were treated with the histone deacetylase inhibitor Trichostatin A (TSA; 100 ng/mL) for 24 h. After cell lysis, chromatin was immunoprecipitated with anti-SET antibody and PCR was performed using primers that recognize the promoter of *HIF-1A* and *NFATC3*. INPUT samples consist of total DNA; SET lanes refer to DNA immunoprecipitated with anti-SET antibody. (**D**) Western blot assay using antibodies against acetyl-H2B, acetyl-H3, SET, and β-actin was performed with total proteins from the respective cells treated with TSA (100 ng/mL) for 24 h.

SET binds to hypoacetylated histones and interacts with histone deacetylases, inhibiting the access of histone acetyltransferases to chromatin [13]. In this context, we assessed by Western blot whether SET accumulation changes the H2B and H3 acetylation levels (Figure 7A). Consistent with our previous report that SET decreases histone H4 acetylation [23], SET accumulation lowered both H2B and H3 acetylation levels, while SET knockdown elevated H2B and H3 acetylation; it suggests a mechanism of gene expression regulation mediated by SET that involves histone acetylation. As SET associates with class I and II histone deacetylases (HDACs) at deacetylated promoters to prevent histone acetylation and arrest gene expression [24], next, we used an immunofluorescence assay to assess the effects of SET accumulation on histone deacetylase 1 (HDAC1). As expected, SET accumulation and SET knockdown were associated with augmented and diminished HDAC1 levels, respectively (Figure 7B).

To demonstrate the role that SET protein plays in the promoter silencing through its direct interaction with hypoacetylated histones, we selected the pharmacological HDAC inhibitor, TSA (Trichostatin A), to increase histone acetylation levels [27]. After SET overexpression, HEK293 cells were treated with TSA, and a ChIP assay was performed to evaluate the capability of SET-promoter interaction (Figure 7C). We analysed the promoter of *HIF-1A* and *NFATC3*, which were downregulated by the SET-promoter interaction (Figure 6C). For both genes, TSA decreased the SET-promoter interaction (Figure 7C - black arrow). Western blot analysis of the efficacy of the TSA treatment demonstrated that it elevated the levels of acetylated histones H2B and H3, and remarkably diminished the SET protein levels (Figure 7D).

### Epigenetic control of gene expression mediated by SET accumulation is more effective through mechanisms involving histone acetylation

**Figure 8:**
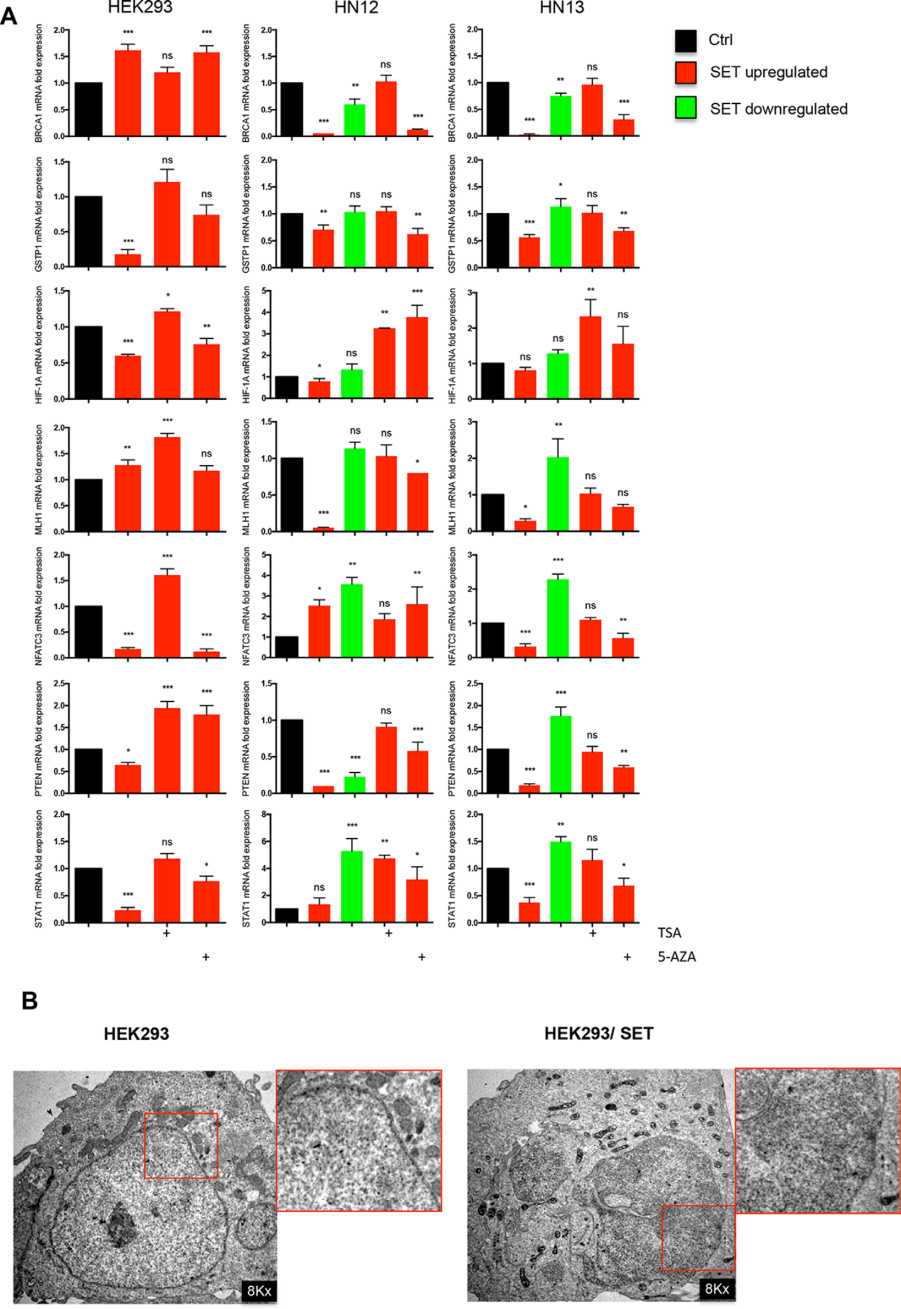
Histone acetylation is more effective than DNA demethylation in reactivating gene expression in SET-accumulating cells (**A**) Quantitative real time PCR was performed using cDNA from HEK293, HEK293/SET, HN12, HN12siSET, HN13, and HN13siSET cells treated with 100 ng/mL TSA (histone deacetylase inhibitor) or 10 μM 5-aza-2'-deoxicytidine (DNA methyltransferase inhibitor). *BRCA1*, *MLH1*, and *PTEN* were assessed through TaqMan probes, and *GSTP1*, *HIF-1A*, *NFATC3*, and *STAT1* through SybrGreen primers. Graphics represent relative quantification of experiments performed in triplicate, calculated using the 2^−ΔΔCt^ method. CDNA from HEK293 cells was used as calibrator; GAPDH and β-globin primers were used as housekeeping. *(*p* < 0.05), **(*p* < 0.01), ***(*p* < 0.001) and ns (non-significant). (**B**) Transmission electron microscopy was performed in HEK293 and HEK293/SET cells to analyze chromatin compaction.

As demonstrated above, SET accumulation promotes DNA hypomethylation through a mechanism of active DNA demethylation involving increased TET1 and 5-hydroxymethylcytosine generation. Controversially, SET decreased histone acetylation levels and expression of transcription factor genes. DNA methylation and histone acetylation are the most studied epigenetic modifications in the context of gene expression. Methylation of gene promoters may trigger deacetylation of histones and hypoacetylated histones may sensitize to targeted DNA methylation [28]. In this sense, we assessed the relationship between SET protein and gene expression using two pharmacological agents, 5-aza-2′-deoxycytidine (5-AZA) and TSA, to inhibit DNA methylation and activate histone acetylation, respectively. We performed qPCR in HEK293, HEK293/SET, HN12, HN12siSET, HN13, and HN13siSET cells to determine how the loss of methylation and gain of acetylation affect the expression of genes previously identified as being regulated by SET protein. By using a relative quantification method [29], we assessed the potential of pharmacological agents to restore the gene expression levels close to those observed in cells without SET accumulation. In general, TSA was more effective than 5-AZA to reverse the effect of SET protein accumulation on gene expression (Figure 8A). Therefore, SET accumulation likely represses gene expression through a histone hypoacetylation-associated mechanism.

Regulation of gene expression depends on histone modifications, such as histone acetylation/methylation, DNA methylation, chromatin remodeling enzymes and effector proteins that, in association, influence the structure and function of chromatin [30]. In addition, evidence suggests an interplay between DNA methylation and histone deacetylation in the silencing of gene transcription [28]. Given that our results indicated that SET accumulation promotes DNA demethylation and histone deacetylation, as well as downregulation of genes, we assessed whether these epigenetic modifications, promoted by SET accumulation, could reflect modifications in chromatin organization. Transmission electron microscopy (TEM) images revealed that SET-accumulating HEK293 cells presented an increase in electron-dense material scattered by the nucleus, which was compatible with chromatin structure, despite exhibiting a loss of the heterochromatin associated with the nuclear lamina (Figure 8B). These results may explain how SET regulates gene expression levels though histone deacetylation and chromatin condensation: together, they could be promoting the repression of transcription.

## DISCUSSION

SET protein, which was first described as stimulating DNA replication and transcription of the adenovirus genome protein [31], has been associated with multiple actions involved in chromatin remodeling and transcription control [10, 13, 32–34]. A SET function linking epigenetic modifications and gene expression has not yet been described. In this context, and considering that SET accumulates in HNSCC [9, 15], we assessed whether SET accumulation could interfere with epigenetic and gene expression control to contribute to cancer development. Here, we demonstrated that SET accumulation is associated with loss of DNA methylation, apparently through active DNA demethylation signaling and, remarkably, reduced methylation levels seem not to directly correlate with gene expression control.

HNSCC is a heterogeneous disease in which DNA methylation status changes according to clinical, environmental, and genetic factors [35]. DNA global hypomethylation has been reported in HNSCC and has been associated with tumour progression, regardless of the methylation status of the gene promoter regions [36]. As expected, we found that three HNSCC cell lines exhibited diminished DNA methylation when compared with normal oral keratinocyte spontaneously immortalized (NOK-SI) and HEK293 cells. SET protein knockdown through RNA interference recovered the DNA methylation status, which was accompanied by augmented 5-methylcytidine levels, indicating that SET participates in the control of global methylation by promoting loss of methylation in HNSCC. However, loss of methylation was not followed by increased expression of the genes analysed. According to TEM images, loss of methylation resulting from SET accumulation may be associated with either reactivation of elements of peripheral heterochromatin, repetitive elements or oncogene activation. These are factors known to favour carcinogenesis [37, 38].

Loss of DNMT1 leads to progressive DNA demethylation through a passive mechanism [26, 39]. It is worth to note that SET accumulation increased both DNMT1 protein levels and enzymatic activity. This finding suggests that the DNA demethylation mechanism associated with SET accumulation is not mediated by DNMT1 loss in a passive manner [40]. DNMT1 is the major enzyme that maintains the cellular DNA methylation levels [41] but, in tumour cells, DNMT1 up-regulation is associated with cellular transformation in tumours presenting both global hyper- or hypomethylation [42–44].

One explanation for the loss of methylation promoted by SET involves the active DNA demethylation process, based on TET proteins that successively oxidize 5-methylcytosine to 5-hydroxymethylcytosine, 5-formylcytosine, and 5-carboxylcytosine, which in turn can be removed from DNA through a base excision repair mechanism [40, 45]. Accordingly, we demonstrated that SET accumulation is associated with a rise in TET1 and 5-hydroxymethylcytosine levels, suggesting the involvement of SET in the active DNA demethylation through accumulation of TET1, which in turn drives 5-hydroxymethylcytosine formation.

Our initial hypothesis that global loss of methylation would result in increased expression of all studied genes was not confirmed; in fact, SET protein downregulated most of the genes analyzed. Recent studies have demonstrated that specific histone modifications can mediate gene repression in cancer cells in the gene promoter region through a DNA methylation state-independent mechanism [46–48]. In addition, it has been proposed that, rather than a mechanism to stably lock gene expression, DNA methylation works as a marker to maintain gene silencing [49, 50]. Surprisingly, SET accumulation did not increase the expression of the hypomethylated genes analyzed, suggesting that SET regulates gene expression through a mechanism independent of DNA methylation of the gene promoter regions. Given that SET accumulation inhibits histone acetylation [13], we assessed whether SET negatively regulates gene expression by controlling the histone acetylation process.

Lysine acetylation in histone tails relaxes the chromatin structure and favours gene transcription [51, 52]. SET protein was identified in association with hypoacetylated histones, maintaining the gene silencing [23, 24]. Here, we found that SET accumulation lowered the expression of most of the genes analysed, regardless of DNA methylation state. Given that histone deacetylase inhibitors can reactivate gene expression without loss of methylation [50], we used TSA, a pharmacological HDAC inhibitor, to increase histone acetylation and 5-AZA, a demethylating agent, to inhibit DNA methylation. By exploring the gene expression levels, our results demonstrated that TSA was more effective than 5-AZA in recovering the gene expression close to basal levels, suggesting that *(i)* the main mechanism of gene transcription silencing involves histone hypoacetylation and chromatin compacting in SET-accumulating cells, and *(ii)* this mechanism is independent of the DNA methylation status.

This study provides evidence supporting that SET accumulation is important for tumorigenesis in HNSCC. SET would regulate the expression of several genes associated with cell differentiation, DNA damage repair, and development that we investigated, such as transcription factors and tumour suppressors (*STAT1, STAT3, NFkB, NFATC3, MLH1, PTEN, BRCA1, HIF1A, ATF1*, and *CREB*). Furthermore, DNA demethylation, associated with SET accumulation, may also favour HNSCC tumorigenesis by, for example, reactivating heterochromatin elements as repetitive sequences and oncogenes.

Overall, SET accumulation is implicated in epigenetic modifications, such as DNA hypomethylation and histone hypoacetylation. It is associated with gene expression regulation and chromatin organization, thereby affecting gene expression control, DNA damage repair, and genome instability. Certainly, SET protein activity can contribute to HNSCC development. We propose HDAC inhibitors as potential pharmacological agents to recovery gene expression in SET-accumulating tumours.

## MATERIALS AND METHODS

### Cell lines and transfection

Head and neck squamous cell carcinomas (HNSCCs) HN6, HN12, and HN13 [53], HEK293 (human embryonic kidney; ATCC, Manassas, VA, USA) and NOK-SI (normal oral keratinocytes) [54] cell lines were cultured in DMEM supplemented with 10% fetal bovine serum, penicillin (100 U/ml), and streptomycin (100 mg/ml) (Sigma-Aldrich, St. Louis, MO, USA), at 37°C, in a humidified atmosphere containing 5% CO_2_.

For assays using SET overexpression, HEK293 and NOK-SI cells were transfected with SET DNA construction using the PolyFect Reagent (Qiagen, Valencia, CA, USA) following the manufacturer's instructions: HEK293/SET and NOK-I/SET, respectively. A *SET* human full-length cDNA clone (pCMV SPORT6; NM_003011.3) was purchased from Invitrogen and transferred to a pcDNA3.1 vector (Invitrogen - Thermo-Fisher Scientific - Waltham, MA, USA). Negative control cells were transfected using a pcDNA3.1 empty vector.

For assays using SET knockdown, HNSCC cell lines were transfected with double-stranded RNA oligonucleotides directed against *SET* (GS6418; Qiagen, Valencia, CA, USA) using HiPerFect Transfection Reagent (Qiagen, Valencia, CA, USA) following the manufacturer's instructions: HN6siSET, HN12siSET, and HN13siSET, respectively. The si*CONTROL* AllStars siRNA Negative Control (Qiagen, Valencia, CA, USA) was used as a negative siRNA control.

### DNA methylation analysis

Genomic DNA was isolated from cell lines through digestion with proteinase K followed by phenol-chloroform extraction and ethanol precipitation. Genomic DNA (1 μg) from HNSCC (control or with SET knockdown), HEK293 and NOK-SI (control or with SET overexpression) cells were digested using the EpitTect methyl DNA restriction Kit (Qiagen, Valencia, CA, USA) according to the manufacturer's instructions, and tumour suppressor gene EpiTect methyl qPCR Arrays (Qiagen, Valencia, CA, USA) were applied.

### Analysis of 5-methylcytidine and 5-hydroxymethylcytosine levels by using flow cytometry

The protocol was performed as previously described [55]. Briefly, cells were washed twice with phosphate buffered saline (PBS) pH 7.4 supplemented with 1% bovine serum albumin (BSA) and 0.1% Tween 20 (PBST-BSA), and fixed with 0.25% paraformaldehyde. Cells were maintained at 4°C for 10 min before addition of 9 vol of methanol/PBS. Then, cells were treated with 2 N HCl at 37°C and with 0.1 M borate buffer pH 8.5. Samples were treated with RNase (10 mg/mL), blocked with PBST-BSA and incubated with anti-5-methylcytidine (sc-56615 - Santa Cruz Biotechnology, Santa Cruz, CA, USA) or 5-hydroxymethylcytosine (ab214728 - Abcam, Cambridge, MA, USA). After incubation with TRITC-conjugated secondary antibody for 1 h, cells were analysed using Guava easyCyte^™^ (Millipore, Billerica, MA, USA) cytometer.

### RNA extraction and real time PCR

RNA was isolated from cell lines using TRIzol reagent (Invitrogen, Carlsbad, CA, USA), according to the manufacturer's protocol. RQ1 RNase-Free DNase (Promega, Madison, WI, USA) was used to treat RNA. cDNA synthesis was performed using a SuperScript III Reverse Transcriptase (Invitrogen, Carlsbad, CA, USA) protocol.

The mRNA sequences were obtained from NCBI database (www.ncbi.nlm.nih.gov), and primers (Supplementary Table 1) were constructed using the Gene Runner program (version 3.05, Hastings Software, Inc.). Reactions were performed in a medium (10 μL) containing 5 μL Fast EvaGreen Master Mix (Uniscience, Cambridge, U.K.), 0.3 μM per primer, and 100 ng cDNA in a Mastercycler ep-Realplex thermocycler (Eppendorf, Hamburg, Germany). *BRCA1* (Hs01556193_m1), *MLH1* (Hs00979919_m1) and *PTEN* (Hs02621230_s1) mRNA levels were assessed by TaqMan assay (Thermo Fisher Scientific, Waltham, MA, USA) according to the manufacturer's protocol.

### Immunofluorescence assay

Cells were placed on glass coverslips in 24-well plates and fixed with methanol for 6 min, at −20°C. In addition, 0.5% (v/v) Triton X-100 in PBS and 3% (w/v) BSA was used for blocking. Primary antibodies anti-DNMT1 (#D59A4) and HDAC1 (#10E2) (Cell signaling, Danvers, MA, USA) and anti-TET1 (#sc-163443 - Santa Cruz Biotechnology, Santa Cruz, CA, USA) were incubated overnight. After incubation with an Alexa Fluor 546-, FITC- or TRITC-conjugated secondary antibody for 1 h, the cells were stained with DAPI (Sigma-Aldrich, St Louis, MO, USA) and visualized using a Zeiss Axiovert 40 CFL Microscope and Zeiss AxioVision 4.8.2 software (Munich, German).

### DMNT activity assay

Protein fractions were obtained using ProteoJET™ Cytoplasmic and Nuclear Protein Extraction Kit (Fermentas - Thermo-Fisher Scientific - Waltham, MA, USA), according to the manufacturer's instructions. Nuclear fractions were subjected to a DNA methyltransferase activity assay using EpiSeeker DNMT Activity Quantification Kit (Abcam, Cambridge, MA, USA) according to the manufacturer's protocol.

### Human transcription factor PCR array

Total RNA was isolated from cell lines using TRIzol reagent (Invitrogen, Carlsbad, CA, USA). Complementary DNA was synthetized using an RT^2^ first strand kit (SABioscience - Qiagen, Valencia, CA, USA). Real time PCR was performed using an RT^2^ Profiler PCR array system (SABioscience - Qiagen, Valencia, CA, USA) with SYBR Green PCR Master Mix, according to manufacturer's instructions, in a Mastercycler ep-Realplex thermocycler (Eppendorf, Hamburg, Germany).

### Luciferase reporter assay

The protocol was performed as previously described [56]. Briefly, HEK293 cells were transfected with SET pcDNA3.1 and pGL3-NFκB-*Luc* or pGL3-STAT3-*Luc* (kindly provided by Dr. Squarize) [56] promoter reporter constructs containing firefly luciferase cDNA, and pRL-null normalization construct containing *Renilla* luciferase from *Renilla reniformis*. Firefly and *Renilla* luciferase activity were measured in the cellular extract using Dual-Glo^®^ Luciferase Assay System (Promega, Madison, WI, USA), 24 h after transfection in a luminometer (AutoLumat LB 953, Berthold).

### Chromatin immunoprecipitation assay (ChIP)

ChIP assay was performed as previously described [23]. Cells were cross-linked with 1% formaldehyde and then lysed. The DNA-protein complexes were immunoprecipitated with anti-SET (Santa Cruz Biotechnology, Santa Cruz, CA, USA) and protein G agarose beads (GE HealthCare, Piscataway, NJ, USA). DNA was extracted with phenol/chloroform post-reversing of the DNA-protein crosslinks. PCR reaction was performed using the sequence primers for *GSTP1*, *HIF-1A*, *NFATC3*, *NFkB* and *PTEN* (Supplementary Table 2). PCR products were analysed using 1.5% agarose gel.

### Immunoblotting assay

Cultured cells were harvested and sonicated, and protein concentration of the cellular extract was determined through DC protein assay (Bio-Rad, Hercules, CA, USA). Aliquots of total protein (30–50 μg) from each sample were resolved by sodium dodecyl sulphate (SDS) PAGE and transferred to a PVDF membrane. The membrane was incubated overnight with primary antibodies for ATF1 (#ab208436 - Abcam, Cambridge, MA, USA), CREB (#9197), acetyl-H2B (Lys20) (#2571) acetyl-H3 (Lys9) (#9649), Histone H3 (#9715), p65/NFkB (#3034), NFATC3 (#4998), STAT1 (#9172), STAT3 (#12640) (Cell signaling, Danvers, MA, USA), SET (sc-133138) and β-actin (sc-47778) (Santa Cruz Biotechnology, Santa Cruz, CA, USA). The membrane was washed and incubated with secondary antibodies conjugated with horseradish peroxidase for 1 h. Bound antibodies were detected using an ECL Western blotting system (GE Healthcare, Little Chalfont, UK).

### Transmission electron microscopy

HEK293 and HEK293/SET cells were collected after trypsin digestion, washed in fresh PBS (pH 7.4), fixed by immersion in glutaraldehyde, post-fixed in osmium tetroxide, stained in block with uranium acetate, dehydrated, and embedded in resin (Embed 812, EM Sciences). Ultrathin sections (60 nm) were collected on Pioloform (Ted Pella, Redding, CA) and carbon-coated single sloth grids and contrasted with uranyl acetate and lead citrate. Cells were examined on a ZEISS LEO 906 electron microscope.

### Statistical analyses

Statistical analyses were performed using GraphPad Prism software (version 5.0, USA). Student's *t-test* or ANOVA was used to examine the association between media and treatments. *P* values < 0.05 were considered significant.

## SUPPLEMENTARY MATERIALS TABLES


