# Supplementary material for: SET oncoprotein accumulation regulates transcription through DNA demethylation and histone hypoacetylation
Source: Oncotarget. 2017 Mar 1;8(16):26802–18. doi: 10.18632/oncotarget.15818 (PMC5432298; doi:10.18632/oncotarget.15818)
Supplement: Supplementary file 1 [file oncotarget-08-26802-s001.pdf]

SET oncoprotein accumulation regulates transcription through DNA demethylation and histone hypoacetylation

Supplementary Materials

Supplementary Table 1: Primers of real time PCR assay

GENE	*SEQUENCE (5'-3')*	*SIZE (bp)*
ATF2 (F)	ATGGTAGCGGATTGGTTAGG	120
ATF2 (R)	TTTGGGTCTGTGGAGTTGTG	
ATF3 (F)	GCCCCTGAAGAAGATGAAAG	138
ATF3 (R)	TTCAGCATTCACACTTTCCA	
β-GLOBIN (F)	GCCTCGCTGTCCACCTTCCA	124
β-GLOBIN (R)	AGAAAGGGTGTAACGCAACTAAG	
CTNNB1 (F)	GTATGAGTGGGAACAGGGATTT	117
CTNNB1 (R)	GTCTCAGGGAACATAGCAGC	
GAPDH (F)	GACTTCAACAGCGACACCCACTC	126
GAPDH (R)	GTCCACCACCCTGTTGCTGTAG	
GSTP-1 (F)	GCAAATACATCTCCCTCATCTACAC	165
GSTP-1 (R)	AGCAGGTTGTAGTCAGCGAAGGAG	
HIF-1A (F)	GCCGAGGAAGAACTATGAACA	152
HIF -1A (R)	AATGGGTTCACAAATCAGCA	
MYB (F)	GGCACACAAGAGACTGGGGA	155
MYB (R)	CTGGCTGGCTGGCTTTTGAA	
NFATC3 (F)	GCCACGCCGATGACTACTGC	134
NFATC3 (R)	TGCACAATCATCTGGCTCAAG	
RELA (F)	GAATCCAGTGTGTGAAGAAGC	122
RELA (R)	CACAGCATTCAGGTCGTAGTC	
STAT1 (F)	TAGTGGAGTGGAAGCGGAGA	162
STAT1 (R)	CGTAGGTGTATTTCTGTTCCA	

Supplementary Table 2: Primers of chromatin immunoprecipitation (ChIP) assay

GENE	*SEQUENCE (5'-3')*	*SIZE (bp)*
GSTP-1 (F)	CCTCTCCCCTGCCCTGTGAAG	380
GSTP-1 (R)	GGCGAAACTCCAGCGAAGGC	
HIF-1A (F)	GAAAGAGAGCAGGAGCATTACA	290
HIF-1A (R)	AAGTCCCCACCCCAGCCCAC	
NFATC3 (F)	GGAGACCGATAACCCTGTGTGT	284
NFATC3 (R)	AATAGATGAAAAACCCAGTGCG	
NFkB1 (F)	CGTGGGAGGAGGTTGACAGTAG	264
NFkB1 (R)	CCAACCACCTTCCCTCTCTCCT	
PTEN (F)	GAGGGGAAAGATGCTCGACT	232
PTEN (R)	GAGGCGAGGATAACGAGCTA	
